# Development of a Highly Specific RPA/CRISPR-Cas13a Assay for Detection of *Pseudomonas aeruginosa* Virulence Factor ExoU in Blood Samples

**DOI:** 10.3390/cimb48060551

**Published:** 2026-05-24

**Authors:** Lucía Ceballos-Romero, Soraya Herrera-Espejo, Daniel Atassi, Pilar Sánchez-Suero, Jerónimo Pachón, José Miguel Cisneros, María Eugenia Pachón-Ibáñez

**Affiliations:** 1 Centro de Investigación Biomédica en Red (CIBER) de Enfermedades Infecciosas (CIBERINFEC), Instituto de Salud Carlos III, 28029 Madrid, Spain; lceballos-ibis@us.es (L.C.-R.); mpsanchez-ibis@us.es (P.S.-S.); josem.cisneros.sspa@juntadeandalucia.es (J.M.C.); mpachon-ibis@us.es (M.E.P.-I.); 2 Clinical Unit of Infectious Diseases, Microbiology and Parasitology, Institute of Biomedicine of Seville (IBiS), Virgen del Rocio University Hospital/CSIC/University of Seville, 41013 Seville, Spain; 3 Institute of Biomedicine of Seville (IBiS), Virgen del Rocio University Hospital/CSIC/University of Seville, 41013 Seville, Spain; sherrera-ibis@us.es (S.H.-E.); danatassi4@gmail.com (D.A.); 4 Department of Medicine, School of Medicine, University of Seville, 41009 Seville, Spain

**Keywords:** rapid diagnosis, *Pseudomonas aeruginosa*, multidrug-resistance detection, high-specificity, RPA/CRISPR-Cas13a

## Abstract

Rapid detection of *Pseudomonas aeruginosa* and its virulence factor ExoU is essential for improving patient outcomes. In this study, a CRISPR–Cas13a-based diagnostic assay combined with recombinase polymerase amplification (RPA) was developed to detect *P. aeruginosa* and the *exoU* gene in blood samples. The assay demonstrated robust amplification, with detection limits of 6 log_10_ and 8 log_10_ CFU/mL in Luria–Bertani medium and blood, respectively, and a 100% specificity, without cross-reactivity against four Gram-negative bacilli and *Staphylococcus aureus* reference strains. The utilisation of a fluorescence-based readout facilitated unambiguous discrimination between *P. aeruginosa* and *P. aeruginosa*/*exoU^+^* isolates vs. negative controls. In conclusion, these results support the potential of RPA/CRISPR-Cas13a diagnostics for the rapid identification of *P. aeruginosa* and its ExoU virulence factor. Further optimisation and clinical validation are required to confirm its utility as a bedside diagnostic test, where its application would speed up clinical decisions in the treatment of these infections.

## 1. Introduction

*Pseudomonas aeruginosa* is a threat to public health worldwide [1], being among the bacterial species that most frequently cause invasive infections in humans, mostly in persons with comorbidities and in health care-related scenarios. The 2024 update report on bacterial pathogens of public health importance from the World Health Organization (WHO), aimed at guiding research and development and strategies to prevent and control antimicrobial resistance, places *P. aeruginosa* at the top of the list [1]. Bloodstream infections (BSIs) caused by these species are associated with high mortality, ranging from 12% to 60% [2].

The pathogenicity of *P. aeruginosa* is due to a wide range of virulence factors, among which the ExoU effector of the type III secretion system stands out and has been linked to both acute cytotoxicity and adverse outcomes in patients [3]. This virulence trait, combined with both acquired and inherent resistance mechanisms and metabolic versatility, contributes to its intrinsic and acquired resistance to antibiotics [4]. The therapeutic options for these infections are limited by their frequent development of resistance during treatment [2], in addition to the increase in the incidence of multidrug-resistant (MDR) and extensively drug-resistant (XDR) strains in recent years [5]. Furthermore, *exoU-positive* strains demonstrate significantly higher rates of fluoroquinolone and beta-lactam resistance, making rapid virulence genotyping essential for appropriate empirical therapy [6].

Thus, in this clinical context, and in the related search for new alternatives for the treatment of *P. aeruginosa* infections, the identification of the pathogen causing an infection would improve treatment and therefore the final outcome. Currently, in microbiological diagnostic workflows, there are often 48 h between pathogen identification and resistance profiling, leading, on occasion, to inappropriate empirical treatments [7]. These delays have been associated with significantly higher mortality rates compared to those in patients receiving appropriate initial therapy (30% vs. 18%) [8]. Thus, the development of rapid and sensitive diagnostic tools is key to improving early therapy guidance and reducing mortality [9]. Moreover, the rapid detection of *exoU^+^ P. aeruginosa* strains is particularly urgent given their impact on patient outcomes, as they majorly contribute towards alveolar epithelial injury and rapid host cell membrane destruction in *P. aeruginosa* infection [10,11]. The presence of ExoU is independently associated with a nearly two-fold increase in early mortality in bloodstream infections, with survival rates at 5 days of only 31.4% for *exoU^+^* versus 53.2% for *exoU^−^* isolates [5].

**Figure 1:**
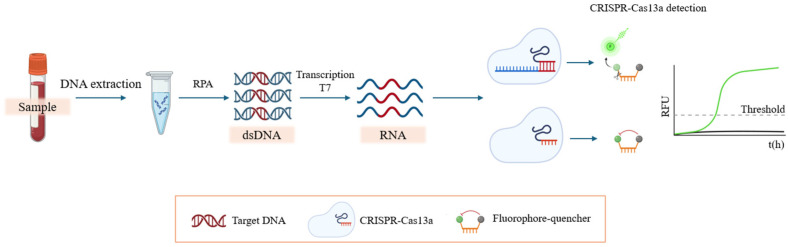
Diagram of the RPA/CRISPR–Cas13a assay.

Current molecular detection methods, such as conventional PCR or loop-mediated isothermal amplification (LAMP), require specialised, robust and expensive equipment, and longer waiting times [12,13]. An alternative diagnostic option is CRISPR systems, which have evolved from genome-editing tools to powerful diagnostic platforms [14,15,16]. The type VI CRISPR–Cas system utilises the Cas13 (formerly C2c2) endonuclease [17], where Cas13a functions as an RNA-guided RNase with an unspecific catalytic function. Cas13a activates its trans-cleavage activity only when crRNA recognises its RNA target sequence, exscinding all nearby single-stranded RNA molecules. By introducing fluorophore-quencher-labelled ssRNA probes into the reaction, this cleavage results in a rapid and exponential amplification of the fluorescent signal (Figure 1) [14,18]. Unlike Cas12-based systems, Cas13a does not require a protospacer adjacent motif (PAM), providing greater flexibility in target selection. Furthermore, although Cas13a-based detection involves a transcription step, this can be integrated with the detection reaction and performed concurrently, minimising its impact on the overall assay time. In addition, the transcription step enables further signal amplification and, together with the strong collateral cleavage activity of Cas13a, supports its suitability for sensitive detection in this study [19]. In 2017, Zhang et al. took advantage of this mechanism to develop a platform called Specific High-sensitivity Enzymatic Reporter Unlocking (SHERLOCK), which combines isothermal pre-amplification with the Cas13a system to achieve a highly sensitive detection technology that requires no expensive equipment and short incubation times [18].

This CRISPR system is used, employing different approaches, to detect resistant bacteria such as *Acinetobacter baumannii* [20]. Regarding the latter, it was used to find putative genes associated with phage resistance, helping to uncover the existence of a wide range of genes associated with phage resistance and their acquisition [20]. Another study, in which a new CRISPR–Cas13-based in vitro diagnostic assay, CRISPR-against Group B *Streptococcus* (GBS), was developed and validated on >400 clinical cases to evaluate and compare different technologies, found that the CRISPR-GBS system is fast and easy to use, with low instrument costs and a higher sensitivity than traditional PCR [21]. No study has developed an RPA/CRISPR-Cas13a assay specifically to detect the *exoU* virulence gene in *P. aeruginosa*.

Thus, our objective was to develop and validate a new rapid, specific and sensitive diagnostic method based on a CRISPR–Cas13 system to identify ExoU, the main virulence factor of *P. aeruginosa*, in blood.

## 2. Materials and Methods

### 2.1. Bacterial Isolates

Seven bacterial spp. were used: two *P. aeruginosa* clinical isolates (MAD02-021 (*exoU*^+^) and MAD02-007 (*exoU*^−^)) from the GEMARA/REIPI study [22] and five reference strains used as negative controls (*Klebsiella pneumoniae* CECT 997, *Acinetobacter baumannii* ATCC 19606, *Escherichia coli* ATCC 25922, *Enterobacter cloacae* ATCC 13,043 and *Staphylococcus aureus* ATCC 25923) (GC Standards, Madrid, Spain).

### 2.2. DNA Preparation and Extraction from Purified Isolates

To evaluate the analytical sensitivity of the recombinase polymerase amplification RPA/CRISPR–Cas13a detection assay in Luria–Bertani (LB, Merck, Madrid, Spain) medium culture, 10-fold serial stirred dilutions of each strain from overnight cultures at 37 °C were prepared in 20 mL of LB. Then, two bacterial concentrations, 8 and 6 log_10_ CFU/mL, were tested for the detection of *oprL* in LB medium cultures of both clinical *P. aeruginosa* strains MAD02-021 (*exoU*^+^) and MAD02-007 (*exoU*^−^) and for the five control strains. DNA was extracted using a QIAamp DNA mini kit (QIAgen; Barcelona, Spain) following the kit protocol [23] and then stored at −20 °C until analysis.

For *P. aeruginosa* detection in blood, serial dilutions of bacterial cultures were grown overnight at 37 °C and stirred in order to obtain bacterial concentrations of approximately 8 and 7 log_10_ CFU/mL. These solutions were then inoculated into 500 µL of blood from healthy volunteers. For DNA extraction, the instructions of the E.Z.N.A.^®^ Universal Pathogen Kit were followed (Omega Bio-tek, Barcelona, Spain) [24].

The study protocol was approved by the Ethics Committee of Virgen Macarena and Virgen del Rocío University Hospitals (Approval Code: 202112016285, Approval Date: 20 January 2021). Informed consent was obtained from healthy volunteers, and the study complied with the principles outlined in the Declaration of Helsinki (1975, revised in 2013).

### 2.3. Guide RNA and Primer Design for P. aeruginosa Detection

The RPA primers (25–35 nt) were designed following the instructions of the TwistAmp^®^ Basic kit (TwistDx Limited; Thermo Fisher Scientific, Madrid, Spain) [25] to obtain an amplification between 100 and 300 bp (Supplementary Tables S1 and S2). Primer specificity was confirmed using NCBI Primer-BLAST V2.5.0 (https://www.ncbi.nlm.nih.gov/tools/primer-blast/, accessed 20 February 2026). The gene encoding peptidoglycan-associated lipoprotein (OprL) was selected as a *P. aeruginosa*-specific detection marker, as it is specific and highly conserved in *P. aeruginosa* strains [26]. A fragment of 287 bp of *exoU* was selected to detect this virulence factor (Supplementary Table S3). Successful amplification and the presence of RPA products were verified by 1% agarose gel electrophoresis performed in a horizontal electrophoresis chamber (Bio-Rad, Hercules, CA, USA). Electrophoresis was carried out in 1× TAE buffer at 100 V for 30 min. RPA was performed in a thermocycler (Bio-Rad, Hercules, CA, USA) at 37 °C for 50 min.

Specific crRNAs targeting the previously mentioned genes were designed using the CHOPCHOP tool V3 (http://chopchop.cbu.uib.no/). This software provides efficient crRNA candidates based on a scoring system [27]; crRNAs were synthesised by Integrated DNA Technologies (IDT, Coralville, IA, USA). The protocol detailed by Kellner et al. was followed for gene detection [27]. The master mix for each reaction contained 4.25 μL of nuclease-free water, 5 μL of rNTPs (New England Biolabs (NEB), Barcelona, Spain), 5 μL of Cas13 10X reaction buffer (MCLAB, San Francisco, CA, USA), 2 μL of LwaCas13a (63.3 μg/mL, MCLAB), 1.25 μL of murine RNAase inhibitor (NEB), 1.5 μL of T7 RNA polymerase (NEB), 1 μL of the corresponding crRNA (10 ng/μL, IDT), and 3 μL of RNaseAlert-1 substrate (IDT). For each reaction, 2 μL of RPA product containing the target nucleic acid was added. The fluorescence signal was recorded every 5 min for 3 h at 37 °C on a CLARIOstar microplate reader (BMG LABTECH, Ortenberg, Germany) from the Scientific Instrumentation Service of the Institute of Biomedicine of Seville (excitation/emission wavelength: 470 nm/515 nm; dichroic filter: 491.2; filter width: 9.1 nm). A minimum of four replicates were completed for each strain on different days to avoid bias. Moreover, each one of these replicates, per strain analysed, was also performed for each condition, i.e., culture medium or blood.

### 2.4. Evaluation of the Detection Limit and Specificity of SHERLOCK and Determination of the Threshold of Positive Samples

The detection capability of the RPA/CRISPR–Cas13a system was measured by fluorescence, expressed in relative fluorescence units (RFUs) using a microplate reader (CLARIOstar^®^, BMG LABTECH, Ortenberg, Germany), and the results were exported using MARS (version 5.04, Verden, Germany) data analysis software. To assess the limit of detection (LOD), two different bacterial culture concentrations were evaluated—8 and 6 log_10_ CFU/mL—in LB medium and in blood. Fluorescence values were calculated as the difference between the different time points measured for each condition during the three hours of the assay. Background correction was applied by subtracting the corresponding blank control (medium or blood without bacteria, depending on the assay). If negative values were obtained, they were interpreted as signals below the detection limit and were given a value equal to 0, as they did not represent significant fluorescence. A minimum of four replicates were completed for each strain on different days to avoid bias. Moreover, each one of these replicates, per strain analysed, was also performed for each condition, i.e., culture medium or blood.

**Limit of detection**. To estimate the initial bacterial load that CRISPRCas13a technology was able to detect, serial dilutions of bacterial suspensions of different concentrations were inoculated into blood and LB broth. Bacterial cultures were quantified and adjusted to an initial concentration of 8 log_10_ CFU/mL, and ten-fold serial dilutions were prepared in the corresponding matrices. Finally, 500 µL aliquots of each dilution were used to calculate the LOD. DNA extraction was performed from each dilution using the appropriate extraction kits [23,24]. Subsequently, amplification reactions were carried out using 5 µL of extracted DNA per reaction.

**Statistical analysis** was performed using GraphPad Prism version 9.0 (GraphPad Software, San Diego, CA, USA). To determine whether samples were positive or negative, a fluorescence threshold was established based on the distribution of negative control values. Assuming a normal distribution of fluorescence signals from negative samples, the threshold was defined as the mean fluorescence value of the negative controls plus three times the standard deviation (mean + 3 SDs), representing the upper limit of background signal. According to the empirical rule, this threshold corresponds to a 99.7% confidence interval for true negative samples [28].

An independent threshold was calculated for each experimental condition, including each tested bacterial concentration and culture matrix (LB broth or blood). To ensure conservative and robust detection criteria, the final threshold applied in each assay corresponded to the highest upper limit obtained among all negative controls within the same experimental set. Samples with fluorescence values above this threshold were classified as positive, whereas those below the threshold were considered negative.

## 3. Results

Bacterial colonies of *P. aeruginosa* grown on blood agar plates were resuspended in LB medium and blood samples, which were used as detection matrices.

**Figure 2:**
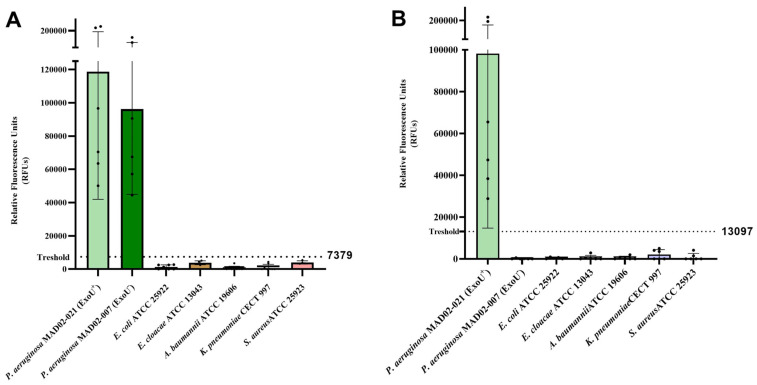
Fluorescence-based detection of *oprL* and *exoU* from clinical *Pseudomonas aeruginosa* isolates and five negative control strains in LB medium. (**A**) Detection of *oprL* in *P. aeruginosa* clinical isolates MAD02-021 (*exoU^+^*) and MAD02-007 (*exoU*^−^) and five negative control strains: *E. coli* ATCC 25922, *E. cloacae* ATCC 13043, *A. baumannii* ATCC 19606, *K. pneumoniae* CECT 997 and *S. aureus* ATCC 25923. (**B**) Detection of *exoU* in *P. aeruginosa* MAD02-021 (*exoU^+^*) and MAD02-007 (*exoU*^−^) and the five negative control strains. The dashed line represents the detection threshold of the technique (mean of negative controls + 3 SD), which corresponds to a 99.7% confidence interval according to the rule of thumb [28]. The error bars represent the SD of the different tests, performed on two different days.

Firstly, to validate the CRISPR–Cas13 detection ability, the recognition of the *oprL* gene, as a *P. aeruginosa*-specific constitutive diagnostic marker in LB, was carried out. A threshold fluorescence limit of 7379 RFUs was established by calculating the upper limit of the highest negative control value. The fluorescence signals obtained for *P. aeruginosa* clinical isolates MAD02-021 (*exoU^+^*) and MAD02-007 (*exoU*^−^) were 93,041 ± 81,321 and 79,497 ± 56,382 RFUs, respectively, confirming positive detection of both isolates. Regarding the five negative control strains, the fluorescence signals ranged from 448 to 4019 RFUs, all below the established threshold limit, confirming the absence of false positives (Figure 2A). For the detection of the ExoU virulence factor in LB medium, the threshold limit of *exoU* was set at 13,097 RFUs. The fluorescence detected in the MAD02-021 (*exoU*^+^) clinical isolate was 98,177 ± 83,460 RFUs versus 0 ± 2071 RFUs for the MAD02-007 (*exoU*^−^) isolate and 0 to 505 RFUs for the five negative control strains; in both latter cases, these values were below the defined threshold limit (Figure 2B, Table 1).

**Figure 3:**
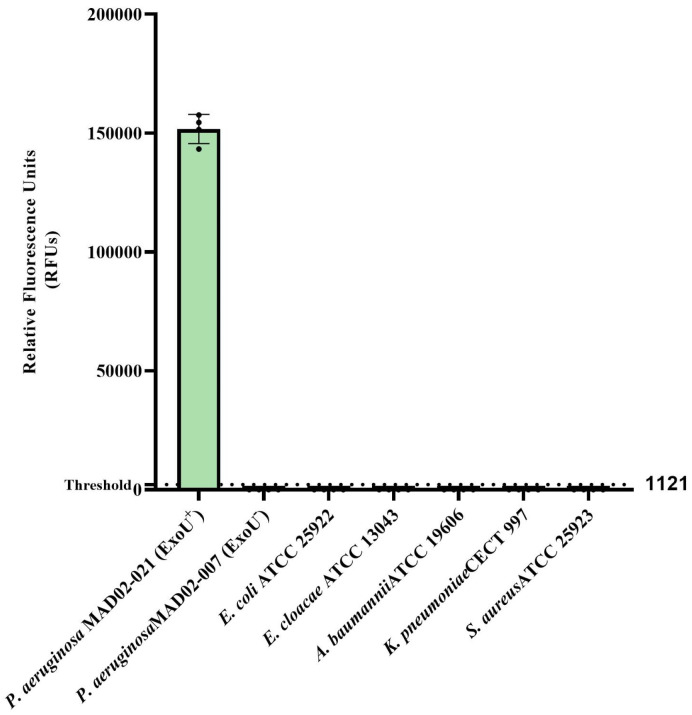
Fluorescence-based detection of *exoU* from clinical *Pseudomonas aeruginosa* isolates and five negative control strains in blood. Detection of *exoU* in *P. aeruginosa* clinical isolates MAD02-021 (*exoU^+^*) and MAD02-007 (*exoU*^−^) and five negative control strains: *E. coli* ATCC 25922, *E. cloacae* ATCC 13043, *A. baumannii* ATCC 19606, *K. pneumoniae* CECT 997 and *S. aureus* ATCC 25923. The dashed line represents the detection threshold of the technique (mean of negative controls + 3 SDs), which corresponds to a 99.7% confidence interval according to the rule of thumb [28]. The error bars represent the SD of the different tests, performed on two different days.

Moreover, *exoU* was detected in bacterial colonies in a blood matrix. The fluorescence signal for the *exoU*-expressing *P. aeruginosa* MAD02-021 strain was 151,688 ± 6152 RFUs, while the detection signals for the non-*exoU*-expressing *P. aeruginosa* MAD02-007 and for the five negative control isolates were 0 ± 167 RFUs and 0 ± 374 RFUs, respectively, both below the detection limit of 1121 RFUs (Figure 3, Table 2).

**Figure 4:**
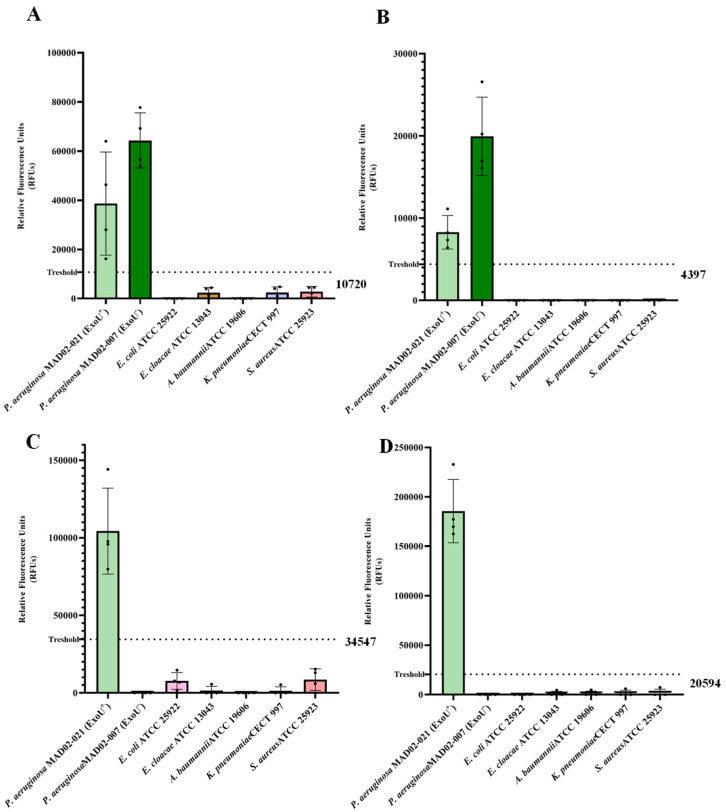
Limit of detection of RPA/CRISPR-Cas13a of *Pseudomonas aeruginosa* and five negative control strains in LB medium. (**A**,**B**) Detection of *oprL* in *P. aeruginosa* MAD02-021 (*exoU*^+^) and MAD02-007 (*exoU*^−^) clinical isolates and five negative control strains—*E. coli* ATCC 25922, *E. cloacae* ATCC 13043, *A. baumannii* ATCC 19606, *K. pneumoniae* CECT 997 and *S. aureus* ATCC 25923—at 8 and 6 log_10_ CFU/mL, respectively. (**C**,**D**) Detection of *exoU* in the *P. aeruginosa* isolates and the five negative control strains at 8 and 6 log_10_ UFC/mL, respectively. The dashed line represents the detection threshold of the technique (mean of negative controls + 3 SDs), which corresponds to a 99.7% confidence interval according to the rule of thumb [28]. The error bars represent the SD of the different tests, performed on two different days.

For *oprL* detection in LB medium, at a concentration of 8 log_10_ CFU/mL, a signal of 38,637 ± 25,078 RFUs and 64,330 ± 30,358 RFUs for *P. aeruginosa* MAD02-021 (*exoU^+^*) and MAD02-007 (*exoU*^−^) isolates was obtained, respectively, both above the established detection threshold of 10,720 RFUs (Figure 4A). At 6 log_10_ CFU/mL signals at 8286 ± 2040 RFUs and 19,962 ± 4761 RFUs for *P. aeruginosa* MAD02-021 (*exoU^+^*) and MAD02-007 (*exoU*^−^) were obtained, respectively, also above the corresponding detection limit of 4397 RFUs (Figure 4B). In contrast, none of the five negative control strains reached the threshold limit, with values between 0 and 2607 RFUs (Figure 4A,B).

For the *exoU* gene LOD in LB medium, at an 8 log_10_ CFU/mL concentration, the fluorescence signal obtained for *P. aeruginosa* MAD02-021 (*exoU^+^*) was 104,305 ± 27,757 RFUs, above the established threshold of 34,547 RFUs. In contrast, *P. aeruginosa* MAD02-007 (*exoU*^−^) exhibited a signal of 0 ± 4563 RFUs, similar to the five negative control strains, ranging from 0 to 7707 RFUs and all below the LOD (Figure 4C). In addition, at a 6 log_10_ CFU/mL concentration, the fluorescence signal for *P. aeruginosa* MAD02-021 (*exoU^+^*) was 185,665 ± 32,031 RFUs, over the threshold of 20,594 RFUs. On the contrary, *P. aeruginosa* MAD02-007 (*exoU*^−^) exhibited a signal of 0 ± 4356 RFUs, which is below the threshold, as were the signals of the five negative control strains, at 0 ± 0 RFUs (Figure 4D). With both crARNs, the assay could detect *exoU* at bacterial concentrations as low as 6 log_10_ CFU/mL, which was defined as the limit of detection under these conditions (Table 3).

**Figure 5:**
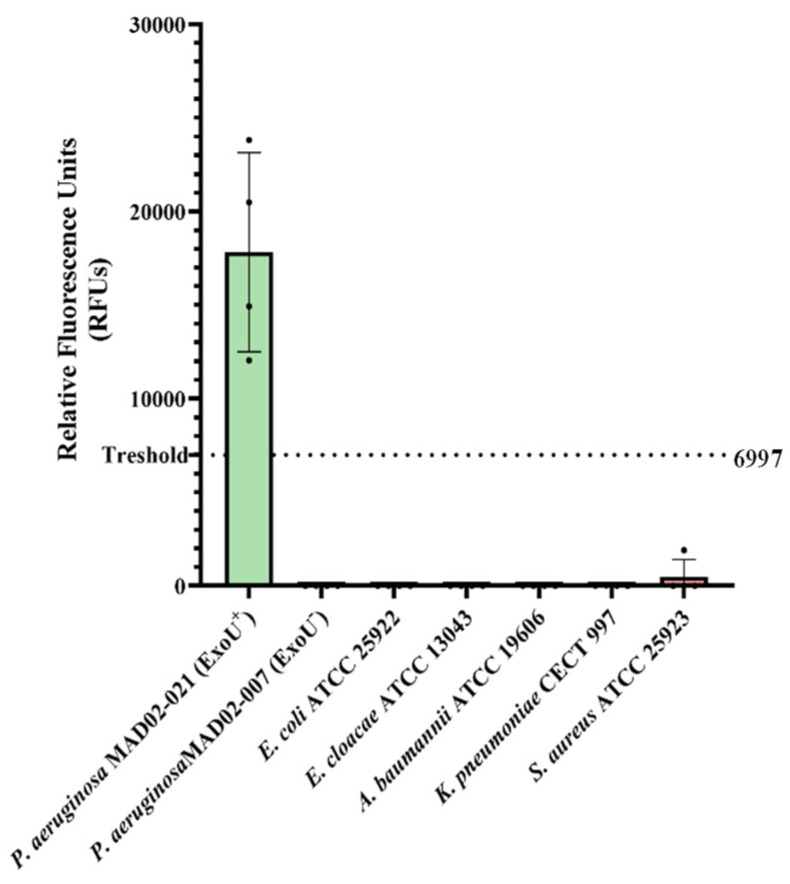
Limit of detection of RPA/CRISPR-Cas13a of *Pseudomonas aeruginosa* and five negative control strains in blood. Detection of *exoU* in *P. aeruginosa* clinical isolates MAD02-021 (*exoU*^+^) and MAD02-007(*exoU*^−^), and the five negative control strains—*E. coli* ATCC 25922, *E. cloacae* ATCC 13043, *A. baumannii* ATCC 19606, *K. pneumoniae* CECT 997, and *S. aureus* ATCC 25923—at a concentration of 8 log_10_ CFU/mL in blood. The dashed line represents the detection threshold of the technique (mean of negative controls + 3 SDs), which corresponds to a 99.7% confidence interval according to the rule of thumb [28]. The error bars represent the SD of the different tests, performed on two different days.

Finally, the analytical sensitivity of the RPA/CRISPR–Cas13a diagnostic assay was assessed in blood for the detection of *exoU* at an 8 log_10_ CFU/mL bacterial concentration. The fluorescence signal was 17,828 ± 5314 RFUs for *P. aeruginosa* MAD02-021 (*exoU*^+^), 0 ± 1246 RFUs for *P. aeruginosa* MAD02-007 (*exoU*^−^), and 0 ± 2332 RFUs for the negative control strains, exceeding the threshold of 6997 RFUs (Figure 5, Table 4).

## 4. Discussion

The recombinase polymerase amplification RPA/CRISPR–Cas13a assay developed in this study allows for rapid qualitative detection (in four hours) of *P. aeruginosa* and its virulence factor ExoU, with no cross-reactivity with common clinical co-pathogens for the detection of *oprL* in LB medium and *exoU* in LB and blood detection matrices. Importantly, the inclusion of the CRISPR–Cas13a system, guided by crRNA, provided additional specificity, allowing for accurate discrimination of target sequences.

In recent years, CRISPR-based diagnostic platforms have become powerful genetic tools for rapid and specific nucleic acid detection. Among these platforms, SHERLOCK (Specific High-sensitivity Enzymatic Reporter Unlocking) has stood out due to its versatility and sensitivity [19]. During the 2020 COVID-19 pandemic, the Food and Drug Administration approved the use of this platform for the detection of SARS-CoV-2 (SHERLOCK™ CRISPR SARS-CoV-2 kit), highlighting its enormous clinical potential [29]. In addition, the SHERLOCK platform has been used to detect a wide range of bacterial pathogens, including *S. aureus*, *A. baumannii*, and *K. pneumoniae*, in clinical samples such as saliva [18,30]. However, to our knowledge, there are no studies that have used it for the detection of *P. aeruginosa* or its virulence factor ExoU directly from blood, only when the pathogen had already been previously isolated from blood cultures [13].

Despite its promising capability, SHERLOCK still faces several technical hurdles that delay its routine clinical implementation. At this time, the main disadvantages of this platform are its off-target effects and false positives (specificity), sometimes due to the high tolerance of Cas enzymes to mismatches [9]. To mitigate the likelihood of off-target effects, in this study, a widely validated optimised design tool, CHOPCHOP, was used for CRISPR-guided RNA design [27]. The use of these tools, together with the possible engineering of Cas proteins, reduces the acceptance of mismatches, improving the specificity and therefore the validity and clinical applicability of SHERLOCK-based diagnostics.

In this study, *P. aeruginosa* LODs of 6 log_10_ and 8 log_10_ CFU/mL were achieved in LB medium and blood samples, respectively. The lower sensitivity observed in blood may be due to the presence of inhibitory components, such as haemoglobin and immunoglobulin G, which are known to interfere with the performance of commonly used molecular assays such as RT-PCR [31]. Several studies have reported lower LODs for SHERLOCK assays; however, these values were often derived from assays using synthetic DNA templates or bacterial isolates, rather than clinical samples. Such approaches employed synthetic *K. pneumoniae* gene fragments [13,32] or isolates of *S. aureus*, *K. pneumoniae*, *A. baumannii* and *S. mutants* [13,31], for example, which could lead to overestimations of the sensitivity of the assay. This uncertainty highlights the need for further optimisation of SHERLOCK platforms in clinically relevant matrices, such as blood.

Although ExoU has been previously detected using conventional PCR [33], which offers high sensitivity and relatively straightforward primer design, this technique typically requires thermocycling equipment and involves longer processing times. In contrast, the proposed method provides higher specificity, enabling discrimination of single-nucleotide differences within target sequences, which is challenging to achieve using primer-based approaches alone [27]. Additionally, the method operates under isothermal conditions, eliminating the need for complex instrumentation. Therefore, rather than replacing PCR, this approach may serve as a complementary tool, particularly in settings where rapid results or point-of-care applicability are critical.

It should be noted that the fluorescence intensities in this study were not consistently correlated with the bacterial concentration, suggesting that the technique works best as a qualitative diagnostic tool based on detection limits. This observation is consistent with those of other CRISPR-based diagnostic studies [9,34], which have reported that SHERLOCK and similar systems often yield qualitative rather than quantitative outputs. Such behaviour may arise from the binary activation of the endonuclease’s collateral cleavage activity, which produces a threshold-dependent fluorescence signal rather than a result proportional to the target concentration. Additionally, extreme variations in reaction kinetics, enzyme saturation, and the presence of inhibitors in blood samples can contribute to signal non-linearity. This fact highlights a potential limitation of this approach, i.e., the fact that detection is qualitative, making it necessary to optimise the SHERLOCK platforms in blood in order to ensure that the technique works as a quantitative diagnostic tool.

## 5. Conclusions

In conclusion, the SHERLOCK tool appears to be a rapid, sensitive, and specific method that allows for the differential detection of not only *P. aeruginosa* in blood, but also of isolates expressing the ExoU virulence factor. Further studies are needed to optimise the reaction kinetics and reduce detection levels, and the tool should be subsequently clinically validated in order to demonstrate its usefulness as a bedside diagnostic test, which would speed up clinical decisions and improve patient outcomes. The next steps are to improve detection at lower bacterial concentrations, validate it against a large cohort of clinical samples with suspected *P. aeruginosa* infection, and compare the results and times with those reported by our hospital’s microbiology department.

## Figures and Tables

**Table 1:** Fluorescence-based detection of DNA from bacterial colonies in LB medium targeting *oprL* and *exoU* genes.

Bacterial spp.			
*oprL*	Mean	SD	SL
*P. aeruginosa* MAD02-021 (*exoU^+^*)	93.041	81.321	-
*P. aeruginosa* MAD02-007 (*exoU*^−^)	79.497	56.382	-
*Escherichia coli* ATCC 25922	1.272	1.303	5.182
*Acinetobacter baumannii* ATCC 19606	448	1.205	4.064
*Enterobacter cloacae* ATCC 13043	3.805	1.192	**7.379**
*Klebsiella pneumoniae* CECT 997	868	1.868	6.471
*Staphylococcus aureus* ATCC 25923	4.019	990	6.988
***exoU***	**Mean**	**SD**	**SL**
*P. aeruginosa* MAD02-021 (*exoU^+^*)	98.177	83.460	-
*P. aeruginosa* MAD02-007 (*exoU*^−^)	−1.169	2.071	5.045
*E. coli* ATCC 25922	−990	2.489	6.478
*A. baumannii* ATCC 19606	−475	1.535	4.129
*E. cloacae* ATCC 13043	183	1.466	4.582
*K. pneumoniae* CECT 997	505	4197	**13.097**
*S. aureus* ATCC 25923	−279	2736	7.930

SD: standard deviation; SL: upper threshold (mean + 3 SDs). The highest upper threshold (in bold) represents the threshold above which fluorescence signals are considered positive for the presence of the target gene.

**Table 2:** Fluorescence-based detection of DNA from bacterial colonies in blood matrix targeting *exoU* gene.

Bacterial spp.	Mean	SD	SL
*P. aeruginosa* MAD02-021 (*exoU^+^*)	151.688	6.152	-
*P. aeruginosa* MAD02-007 (*exoU*^−^)	−6.475	167	501
*E. coli* ATCC 25922	−6.938	71	214
*A. baumannii* ATCC 19606	−6.896	310	931
*E. cloacae* ATCC 13043	−6.867	44	131
*K. pneumoniae* CECT 997	−6.887	127	381
*S. aureus* ATCC 25923	−6.834	374	**1.121**

SD: standard deviation; SL: upper threshold (mean + 3 SDs). The highest upper threshold (in bold) represents the threshold above which fluorescence signals are considered positive for the presence of the target gene.

**Table 3:** Fluorescence-based detection of DNA from bacterial inoculum grown in LB medium at 8 log_10_ and 6 log_10_ CFU/mL, targeting *oprL* and *exoU* genes.

Bacterial spp.	8 log_10_ CFU/mL			6 log_10_ CFU/mL		
*oprL*	Mean	SD	SL	Mean	SD	SL
*P. aeruginosa* MAD02-021 (*exoU^+^*)	38,637	25,078	-	8286	2040	-
*P. aeruginosa* MAD02-007 (*exoU*^−^)	64,330	30,358	-	19,962	4761	-
*E. coli* ATCC 25922	−1252	1087	3261	−7155	805	2416
*A. baumannii* ATCC 19606	−1459	1368	4104	−6946	1153	3459
*E. cloacae* ATCC 13043	2126	2571	9840	−6610	1020	3059
*K. pneumoniae* CECT 997	2121	2866	**10,720**	−6201	1163	**3488**
*S. aureus* ATCC 25923	2607	2442	9934	−5750	1466	4397
***exoU***	**Mean**	**SD**	**SL**	**Mean**	**SD**	**SL**
*P. aeruginosa* MAD02-021 (*exoU^+^*)	104,305	27,757	-	185,665	32,031	-
*P. aeruginosa* MAD02-007 (*exoU*^−^)	−5906	4563	13,690	−6258	4356	13,069
*E. coli* ATCC 25922	7707	5391	23,881	−6442	3798	11,393
*A. baumannii* ATCC 19606	−3696	1398	4194	−3863	5836	17,507
*E. cloacae* ATCC 13043	−525	4807	14,422	−3228	5245	15,734
*K. pneumoniae* CECT 997	−2096	4942	14,826	−3199	6124	18,372
*S. aureus* ATCC 25923	7388	9053	**34,547**	−2814	6855	**20,564**

SD: standard deviation; SL: upper threshold (mean + 3 SDs). The highest upper threshold (in bold) represents the threshold above which fluorescence signals are considered positive for the presence of the target gene.

**Table 4:** Fluorescence-based detection of DNA from bacterial inoculum grown in LB medium at 8 log_10_ CFU/mL, targeting *exoU* gene.

Bacterial spp.	Mean	SD	SL
	8 log_10_ CFU/mL		
*P. aeruginosa* MAD02-021 (*exoU^+^*)	17,828	5314	-
*P. aeruginosa* MAD02-007 (*exoU*^−^)	−6251	1246	3737
*E. coli* ATCC 25922	−5770	789	2366
*A. baumannii* ATCC 19606	−2905	1911	5733
*E. cloacae* ATCC 13043	−3213	1949	5846
*K. pneumoniae* CECT 997	−2671	2309	6926
*S. aureus* ATCC 25923	−2442	2332	**6997**

SD: standard deviation; SL: upper threshold (mean + 3SD). The highest upper threshold (in bold) represents the threshold above which fluorescence signals are considered positive for the presence of the target gene.

## Data Availability

The original contributions presented in this study are included in the article/Supplementary Material. Further inquiries can be directed to the corresponding author.

## Supplementary Materials

The following supporting information can be downloaded at: https://www.mdpi.com/article/10.3390/cimb48060551/s1.
