# Supplementary material for: Development of a Highly Specific RPA/CRISPR-Cas13a Assay for Detection of Pseudomonas aeruginosa Virulence Factor ExoU in Blood Samples
Source: Curr Issues Mol Biol. 2026 May 24;48(6):551. doi: 10.3390/cimb48060551 (PMC13297620; doi:10.3390/cimb48060551)
Supplement: Supplementary file 1 [file cimb-48-00551-s001.zip › cimb-4286733-supplementary.pdf]

Development of a highly specific RPA/CRISPR-Cas13a assay for detection of *Pseudomonas aeruginosa* virulence factor ExoU in blood samples

Supplementary material.

Material and Methods

Supplementary Table S1. RPA primers for the amplification of *oprL* and *exoU* genes.

Gene	Primer	Sequence (5'→3')	Product size
oprL	*oprL*_Fw	**GAAATTAATACGACTCACTATAGGG**TCTACTTCGAGTACGACAGCTCCGA	241 bp
	*oprL*_Rv	CGCTCTTTACCATAGGAAACCAGTT	
exoU	*ExoU*_Fw	**GAAATTAATACGACTCACTATAGGG**TCTGGATGCGTAGCGATCTGTCCGA	287 bp
	*ExoU*_Rv	GCCCTTTTGGCCTCAGGTATGAG	

In bold is highlighted the sequence of the promoter of the T7 RNA polymerase.

Supplementary Table S2. Specific sequences for the amplification and detection of *oprL* and *exoU*.

Gene	Sequence (5'→3')
oprL	TCTACTTCGAGTACGACAGCTCCGACCTGAAGCCGGAAGCCATGCGCGCTCTGGACGTACACGCGAAAGACCTGAAAGGCAGCGGTCAGCGCGTA<br>GTGCTGGAAGGCCACACCGACGAACGCGGCACCCGCGAGTACAATATGGCTCTGGGCGAGCGTCGTGCCAAGGCCGTTCAGCGCTACCTGGTGCT<br>GCAGGGTGTTTCGCCGGCCCAGCTGGAACTGGTTTCCTATGGTAAAGAGCG
exoU	TCTGGATGCGTAGCGATCTGTCCGAGCACGGCCTTGCGTGTCTCGTCGCGCAATAGGCGTTCTAGGGGCTCAGCCCGCGAATCTATGCGTGGGAGTA<br>CATTGAGCAGCAACTCAGAGAAGCCGCCGATCTTGTTGCCCAAGCCCTTTTTCAGCGATGCGCCGATCTCGCTGCTAATGTGTTGGAACAGCTTCAG<br>CTTCTTGTTCGAGCTGTCGAGCAGCGAAATAAGATCCATCTTGTCGGAAAGGGTCTTGAACGCCGCCGGGCTCATACCTGAGGCCAAAAGGGC

Supplementary Table S3. Sequences of the crRNAs for the CRISPR-Cas13a detection of *oprL* and *exoU*.

Gene	Sequence (5'→3')
oprL	**GAUUUAGACUACCCCAAAAACGAAGGGGACUAAAAC**ACCATAGGAAACCAGTTCCAGCT
exoU	**GAUUUAGACUACCCCAAAAACGAAGGGGACUAAAAC**GTTCCAACACATTAGCAGCGAGA

The direct repeat sequence of *Leptotrichia wadeii* Cas13a (LwaCas13a), which enables the crRNA to bind to the enzyme, is highlighted in bold.
